# Clustering of Uterine Natural Killer Cells Around Uterine Glands in Women with Recurrent Implantation Failure and Recurrent Pregnancy Loss: An Immunohistochemical Study

**DOI:** 10.3390/ijms262010109

**Published:** 2025-10-17

**Authors:** Lenka Lapides, Martin Klein, Ivan Varga, Jaroslav Voller, Pavel Babal

**Affiliations:** 1 Faculty of Medicine, Institute of Histology and Embryology, Comenius University, 811 08 Bratislava, Slovakia; vrbova7@uniba.sk (L.L.); ivan.varga@fmed.uniba.sk (I.V.); 2 Faculty of Medicine, Institute of Anatomy and Anthropology, Riga Stradins University, LV-1007 Riga, Latvia; 3 Faculty of Healthcare Studies, University of Western Bohemia, 301 00 Pilsen, Czech Republic; jvoller@fzs.zcu.cz; 4 Faculty of Medicine, Institute of Pathology, Comenius University, 811 08 Bratislava, Slovakia; pavel.babal@fmed.uniba.sk

**Keywords:** uNK cell clusters, assisted reproductive treatment, recurrent implantation failure, recurrent pregnancy loss, immunohistochemistry

## Abstract

The immunological factor of sterility, specifically the abnormal count and activity of uterine NK (uNK) cells, may represent one of the potential contributors affecting specific subgroups of sterile couples undergoing assisted reproductive treatment (ART). Therefore, the primary purpose of the present paper was to assess uNK cell count. A total of 387 endometrial biopsies from patients with recurrent implantation failure (RIF) or recurrent pregnancy loss (RPL) were analyzed to identify abnormalities in uNK cell count, using immunohistopathological evaluation. ANOVA analysis revealed a strong association with factor 0.161 with *p*-value < 0.01, indicating that higher uNK cell count is associated with the presence of clusters (multicellular aggregates of uNK cells). These results suggest that the formation of clusters and the spatial distribution of uNK cells are significant factors in the context of the aforementioned clinical questions. However, the actual translational potential to clinical practice has not yet been established due to several challenges, namely: 1. the constantly changing definitions and diagnostic criteria for RIF and RPL, 2. varying sampling approaches for uNK cells, and 3. the historical lack of clear differentiation between uterine and peripheral NK cells. When all these issues are resolved, the observed tendency of uNK cells to form clusters will need to be a central focus of future investigations addressing RIF and RPL, thus improving ART outcomes.

## 1. Introduction

Natural Killer (NK) cells are innate immune cells that are essential for controlling viral infections and tumors, including the clearance of virally infected or transformed cells, as well as the production of multiple cytokines and chemokines. Traditionally, NK cells have been considered to be cells with a short lifespan, surviving only 1 to 2 weeks and dying after becoming activated or cytotoxic. This conventional characterization has nowadays been overturned, and recent studies describe NK cells as immune cells capable of antigen-specific proliferation or differentiation into a long-lived cell subset with immunological memory-like functions [1]. A unique subset of NK cells plays a crucial role in early pregnancy. These uterine NK (uNK) cells (also known as endometrial NK cells, endometrial granular cells, or by the eponym Hamperl cells) make up 70% of leukocytes in the decidua, are hormonally regulated, and are temporally and spatially associated with implantation [2]. Bearing comparison with Janus, the ancient two-faced Roman god of beginnings and endings, uterine NK cells also have two “faces”, considering their immunological activity. The first “face” is their critical role in defense against viruses and tumors. Even though uNK cells contain cytolytic molecules, they are less cytotoxic than their circulating counterparts, peripheral NK cells. Their second “face” is crucial in maintaining physiological gestation—uNK cells show critical immunomodulatory functions with the potential to control embryo implantation, trophoblast migration and invasion, regulate vascular spiral artery remodeling, and promote embryonic/fetal growth [3]. Dysregulation of uNK cell activity has been implicated in pregnancy complications, notably recurrent implantation failure (RIF) and recurrent pregnancy loss (RPL) (synonymous terms with RPL found in the literature are recurrent miscarriage and recurrent spontaneous abortion. We will be using RPL as the preferred term from now on) [4].

However, the role of uNK cells is likely not limited to implantation and early placenta formation but also extends to subsequent fetal organ development. Current studies show that maternal immune activation (e.g., by viral infection) triggers uNK (decidual) cells to secrete granzyme B, which crosses the placenta, leading to disruption of fetal neuroimmune homeostasis and abnormal neurodevelopment [5]. Interestingly, the functional aspects of uNK cells can be inferred from their spatial organization: uNK cells form clusters around endometrial glands, which correlate with the number of uNK cells. This behavior, which is significantly underresearched, is further expanded in the article.

Assisted reproductive technologies (ART) have become a mainstream treatment for infertility. Their utilization rate is steadily increasing, not only in high-income countries, representing the birth of up to 8% of children in some countries [6]. Since Louise Brown, the first “test tube baby”, was born in 1978, ARTs have improved dramatically, thanks to advances in understanding the early stages of gestation, improvements in cryopreservation technologies, preimplantation genetic testing, and endometrial receptivity assessment, among others [7].

Despite that, there are still many challenges that remain to be tackled. When factors that ART cannot fully compensate for, like chromosomal anomalies, anatomical abnormalities, or the age-related decline in female reproductive performance, are excluded, there is still a portion of RIF/RPL cases without any evident cause. Most explanations in this regard suggest the involvement of the complex yet only partially understood interplay between the semialogenic embryo/fetus and maternal immune system [8].

Regardless of the low percentage of affected couples—up to 2%—these conditions have a high clinical relevance, yet remain without guideline-directed therapy and therapeutic options based on clinical studies.

The complexity of these diagnoses is reflected in the various definitions provided by individual expert societies, such as the European Society of Human Reproduction and Embryology (ESHRE), the American Society for Reproductive Medicine (ASRM), and the Royal College of Obstetricians and Gynaecologists (RCOG). For instance, RPL is defined as more than two, respectively, three miscarriages depending on the society in question [9].

RIF is a complex clinical condition in reproductive medicine, characterized by the inability to achieve a successful pregnancy despite the transfer of good-quality embryos across multiple in vitro fertilization (IVF) cycles. The absence of a universally accepted definition has led to significant challenges in the field, including confusion, a lack of standardization, and difficulty in comparing research findings. Disparities in definitions, particularly regarding the total number of embryos transferred, often result in statistical inconsistencies and complications in interpreting study outcomes [10]. In our research population, we adhered to clinical guidelines based on the ESHRE criteria. These criteria provide a standardized framework for identifying RIF, defined as the failure to achieve pregnancy after the transfer of at least three good-quality embryos, classified as grade A or B based on morphological criteria, or verified through preimplantation genetic testing (PGT) when applicable [11].

Similarly, the definition of RPL has also evolved with new data and differs across societies (ESHRE, RCOG, and ASRM). Sticking again to the ESHRE definition, RPL is defined as the loss of three or more pregnancies—either consecutive or non-consecutive—before fetal viability, which is defined as gestation before 20 weeks or a fetal weight of less than 500 g. The diagnosis of RPL typically relies on clinically documented pregnancy losses, confirmed through ultrasound or histopathological examination [11].

The main objective of this paper is to investigate the relationship between uNK cell count, as determined by immunohistopathological analysis of endometrial samples, and IVF outcomes. By adopting precise and clinically relevant criteria, we aim to provide a standardized approach to studying RIF and RPL, improving comparability and interpretation of findings across reproductive medicine studies.

## 2. Results

Data collected after analyzing the biopsies provided answers to previously unresolved questions. There was no statistically significant difference in uNK cell count regardless of the clinic site, the physician performing the collection, or whether the sample was obtained in an outpatient setting or under sterile conditions in the operating room. Our earlier studies have already demonstrated consistency between samples collected via pipelle biopsy and curettage, showing that both methods reflect the same informative value, particularly regarding uNK cell count and their activation status [12]. Furthermore, the number of uNK cells showed no significant seasonal fluctuations. Analysis of samples collected across different months and seasons revealed a stable number of uNK cells. Due to the limited number of studies focusing on decreased uNK cell counts, another objective was to investigate the possible association between reduced uNK cell numbers and adverse pregnancy outcomes, given that uNK cells are the most abundant immune cells during decidualization and have a well-documented role in placentation. No significant correlation was observed between low uNK cells (using a reference range of <40 cells/mm^2^ for low uNK cells and >300 cells/mm^2^ for elevated uNK cells) and RPL. 21.4% of patients with RPL showed a low uNK cell count, while 15.9% of patients without RPL also showed a low uNK cell count, resulting in a *p*-value of 0.443. Because a high number of patients were using specific medications, we decided to evaluate the potential impact of these treatments on the number of uNK cells. Assessment of the use of the following medications was performed: L-thyroxine, metformin, intralipid, corticosteroids, aspirin, hormone substitution therapy (HST), low molecular weight heparin (LMWH), and bromocriptine. No correlation was found between long-term use of these medications and uNK cell count. In light of the potential impact of food intolerances on infertility, a possible association between the presence of specific intolerances, such as histamine, gluten, lactose, or casein, and the uNK cell count was evaluated. Again, no significant differences in uNK cell numbers were observed in relation to these intolerances. The potential association between uterine NK cell activation and pregnancy outcomes, including clinical pregnancy rates and miscarriage rates, was investigated; however, no statistically significant correlation was found. All of our statistically evaluated observations are summarized in Table 1.

The sole significant correlation was observed between the occurrence of uNK cell clusters (multicellular aggregates) and the overall number of uNK cells. ANOVA analysis revealed a strong association with factor 0.161 (*p*-value < 0.01), indicating that a higher uNK cell count is associated with the presence of clusters. Table 2 summarizes the average uNK cell count in different age groups.

Out of a total of 387 patients, positive uNK cell clusters were observed in 118 cases (30.5%), including 14 patients (3.6%) with strongly positive clusters. In the age group < 30 years, 9 patients (36.0%) showed positive clusters; in the 30–39 years group, 70 patients (29.7%); and in the ≥40 years group, 39 patients (31.0%). However, no correlation was found between cluster positivity and the age groups 20–29, 30–39, and ≥40 years (*p* = 0.68). These results are summarized in Table 3.

**Figure 1:**
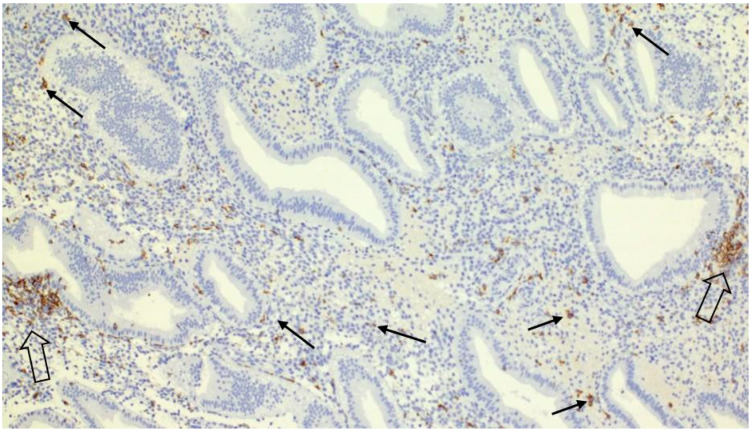
Secretory endometrium infiltrated by uterine NK cells. Uterine NK cells form clusters adjacent to endometrial glands (open arrows) or diffusely infiltrate the stroma as individual cells (arrows)—immunohistochemistry with anti-CD56 antibody, Magnification 100×.

**Figure 2:**
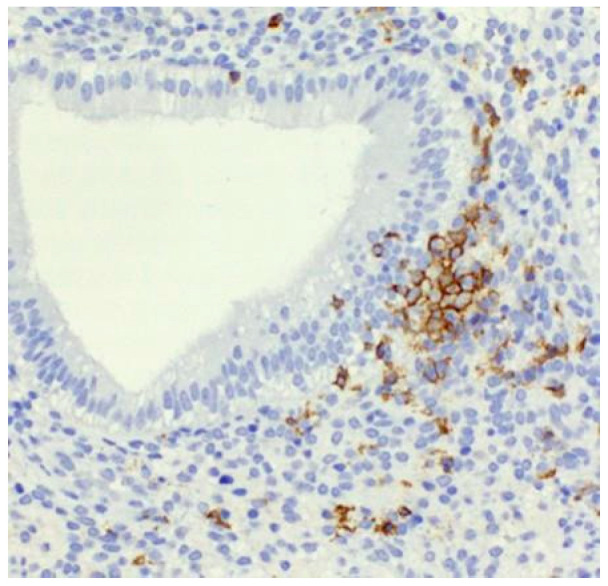
Details on the periglandular uterine NK cells cluster—immunohistochemistry with anti-CD56 antibody, Magnification 400×.

The formation of clusters was visualized by immunohistochemical detection of uNK cell marker CD56 (Figure 1 and Figure 2). As the figures show, these multicellular aggregations of uNK cells resemble B-lymphocyte aggregates, known as lymphoid follicles, and are localized in proximity to uterine glands in the stratum functionale. This finding is unique, considering that previous papers describing uNK cell clusters have mainly focused on animal models. Compared to animal models, human studies describing uNK cell clusters around endometrial glands are rare [13]. Other immune cell populations in the human uterine mucosa, which cluster together, are T cells and a small number of B cells, which form lymphoid aggregates in the stratum basale of the endometrium [14]. On the other hand, in animal models, such as mice, clusters of uNK cells are commonly observed during the secretory phase and early pregnancy, particularly in perivascular locations within the mesometrial lymphoid aggregate of pregnancy (MLAp), a murine-specific lymphoid structure that surrounds the vessels supplying the placenta [15]. This spatial localization suggests a different function. Moreover, in murine models, uNK cells are also embedded in the myometrium of the uterine wall in the form of the mesometrial lymphoid aggregate, a specific feature of murine pregnancy [16,17].

## 3. Discussion

This study investigated the correlation between altered uNK cell count and reproductive outcomes in patients with RIF and RPL. Despite many reports [18,19,20,21,22], as well as our previous hypothesis [23] suggesting a potential role of uNK cell number alteration in influencing implantation and early pregnancy success, our current data did not show a statistically significant association between either elevated or reduced uNK cell counts in the case of RIF or RPL. These findings highlight the complexity of uNK cell biology in fertility, suggesting that cell count alone may not fully reflect their biological or clinical relevance.

The role of uNK cells in reproductive disorders, particularly RIF and RPL, remains widely debated. Many researchers have focused on immune-related causes of implantation failure, as these conditions pose significant medical, emotional, and financial burdens on patients undergoing ART, such as IVF. Given the immunomodulatory function of uNK cells, it has often been hypothesized that dysregulated uNK activity may impair implantation success. However, evidence from the literature is conflicting. A recent meta-analysis conducted by Von Woon et al. [24], which included over 60 studies, reported significantly elevated levels of uNK cells in women with RIF and RPL compared to control groups. In contrast, an earlier meta-analysis by Seshadri and Sunkara [25] found no significant differences in uNK cell count between RPL patients and controls. Such discrepancies underscore significant challenges in uNK research, including heterogeneity in study populations, inconsistent definitions of RIF and RPL, variations in detection methods (immunohistochemistry vs. flow cytometry), a predominance of quantitative over functional studies, a lack of standardized protocols for good-quality embryo evaluation, and variability in both uNK quantification and functional assays. Differences in immunohistochemical techniques and diagnostic criteria further complicate direct comparisons across studies.

Recent research suggests that focusing solely on uNK cell count is insufficient to determine their role in implantation success or failure. Instead, functional characteristics, activation states, and spatial distribution may provide more clinically relevant insights. For instance, Vento-Tormo et al. [26] used single-cell RNA sequencing to characterize distinct uNK cell subsets, demonstrating functional heterogeneity within the population. Similarly, Whettlock et al. [27] investigated the frequency and dynamics of the uNK subset throughout the menstrual cycle and pregnancy, demonstrating that uNK cells undergo phenotypic changes in response to the uterine microenvironment.

While numerous studies have quantified uNK cell counts [21,28,29], far fewer have explored spatial organization or clustering behavior [30]. This study aimed to fill that gap by assessing uNK cell clustering patterns in relation to implantation success and failure. The significant correlation between the number of uNK cells and clustering may offer new insights into the role of uNK cell spatial distribution in reproductive outcomes.

Hazan et al. [31] described that, in the early stages of spiral artery remodeling, leukocytes—including CD56^+^ uNK cells—cluster concentrically around arterioles, with the highest density within 15 μm of the vessel wall, indicating their key role in tissue remodeling, angiogenesis, and immune tolerance. Such perivascular clusters are thought to arise in response to angiogenic and chemokine signals from vascular smooth muscle cells and extravillous trophoblast (EVT). These clusters secrete key vascular endothelial growth factor C (VEGF-C), and placental growth factor (PlGF), key mediators of spiral artery remodeling [27]. Additionally, in murine models, uNK cells have been shown to produce epidermal growth factor (EGF), suggesting a possible role in early embryo development. Using a murine pregnancy model, Kusakabe et al. [32] demonstrated positive immunohistochemical staining for anti-EGF antibodies in uNK cells, with peak expression between days 6 and 9 and day 15 of gestation. This temporal pattern suggests that uNK cells regulate EGF production in a controlled manner. uNK cell clustering can be abnormal in terms of excessive or reduced aggregation, which may reflect pathological conditions. For instance, increased uNK cell density and clustering have been reported in women with RPL, suggesting a potential link to immune dysregulation regarding their spatial organization and functional status [13].

EVT-derived interleukin-6 (IL-6), together with CXCL8, stimulates decidual endothelial cells to secrete the chemokines CCL14 and CXCL6, which in turn promote uNK cell recruitment to these perivascular sites [33,34]. This cytokine–chemokine axis supports the spatial organization of uNK cells observed during the initial stages of spiral artery remodeling.

IL-6 is present in the uterine microenvironment during early pregnancy and, through JAK/STAT signaling, contributes to shaping the cytokine milieu and endothelial chemokine expression, which indirectly influences the recruitment and function of uNK cells. These effects contribute to the interactions between uNK cells and decidual stromal cells, which are crucial for the proper remodeling of the spiral arteries and the successful development of the placenta [35].

Stromal cell-derived factor 1 (SDF-1/CXCL12) plays a crucial role in orchestrating the recruitment of uNK cells in early pregnancy. Invasive trophoblastic cells at the implantation site secrete SDF-1, creating a chemotactic gradient. uNK cells expressing the C-X-C chemokine receptor type 4 (CXCR4) migrate toward higher concentrations of SDF-1, leading to perivascular clustering at key implantation sites. Once clustered, uNK cells secrete cytokines and growth factors that contribute to immune tolerance, tissue remodeling, and fetal–maternal interactions [36,37]. Clustering frequently occurs around signaling centers, extracellular matrix (ECM) components, and blood vessels. Proximity and structural integrity are critical for cell–cell communication and coordinated function.

The role of cell adhesion molecules (CAMs)—especially integrins—is essential for mediating both homotypic and heterotypic interactions within the decidua. In particular, decidual NK cells express integrins that facilitate adhesion to the extracellular matrix and decidual stromal cells, promoting their localization and retention during early pregnancy.

This is consistent with the broader cellular roles of cadherins and integrins in tissue organization and morphogenesis [38,39].

While cadherins are primarily known for their role in epithelial and endothelial adhesion, their direct expression by human uNK cells remains under investigation. Cadherins, particularly E-cadherin and N-cadherin, are expressed by trophoblast and decidual cells and can interact with the killer cell lectin-like receptor G1 (KLRG1) on NK cells. Such interactions may influence the adhesion properties and functional modulation of uNK cells within the decidual environment [40]. The decidual microenvironment, rich in cytokines and growth factors, may regulate cadherin expression, thereby influencing the clustering of uNK cells and tissue remodeling.

uNK cells also express various integrins, which facilitate adhesion and interaction within the uterine microenvironment. CD49a (α1 integrin) and CD103 (αE integrin) are highly expressed on uNK cells, distinguishing them from peripheral blood NK cells [41]. As we have already discussed, the clustering of uNK cells around blood vessels is relatively well-documented, although animal studies are more prevalent than human ones. Even more sparse is the description of uNK cell clustering around endometrial glands, as evidenced by our results. The situation is even more confusing when we consider terminological discrepancies and attempts to define new structures. For example, several studies have attempted to establish a new term, “metrial gland,” in rodents, which is not particularly helpful since it refers to an aggregation of uNK cells rather than a glandular tissue [42,43].

Taken together, this study demonstrates that straightforward uNK cell quantification is arguably insufficient for determining pregnancy outcomes, making this simple approach infeasible for clinical practice in any meaningful manner. However, at the same time, we corroborated the results of previous authors that uNK cell count is related to their clustering patterns and dynamics, which has to be assessed in a complex manner to produce clinically relevant data, predicting the risk of RIF and RPL. Apart from uNK cell count and clustering behavior, it is also necessary to address in more detail the clustering around different structures, mainly blood vessels vs. glands, to elucidate the functional relation of such clustering patterns around individual endometrial components.

## 4. Materials and Methods

We recruited 387 female patients from two Slovak private reproductive medicine clinics, namely the ISCARE, Reproduction Clinic, Gynecology & Urology in Bratislava, and REPROFIT, Reproduction Clinic, Gynecology & Urology in Bratislava. This study was conducted according to the guidelines of the Declaration of Helsinki and approved by the Ethical Committees of both private clinics, where the tissue samples were obtained. Informed consent was obtained from all patients.

Patients had following main factors of sterility: partner with andrological factor (abnormal sperm count and/or abnormal progressive movement), ovarian factor (higher age and/or low level of antimüllerian hormone level < 1 ng/mL), polycystic ovary syndrome, congenital uterine anomalies (e.g., bicornuate uterus), genetic factors, uterine fibroids/leiomyomas, endometriosis (including adenomyosis), endocrine factors (e.g., central hypogonadism) or immunological factor (immunological alteration diagnosed by reproductive immunologist, e.g., elevated NK cells in the peripheral blood or elevated embryotoxic cytokines). The mean age of the included patients was 37 years at the time of examination (range: 23–50 years). The median number of transferred blastocysts per patient was 2. The number of patients who fulfilled the RIF criterion—having three or more embryo (blastocyst quality A or B) transfers was 218. 107 patients with RIF were ≤37 years old. 28 patients fulfilled the criteria for RPL (≥3 pregnancy losses), 16 patients with RPL were ≤37 years old. 17 patients had both diagnoses, RIF and RPL; of these patients, 8 patients were ≤37 years. 158 patients met the criteria for idiopathic sterility. Of these, 82 patients were ≤37 years. In this study, biochemical pregnancies, identified by elevated hCG levels without clinical signs of pregnancy, were also included, as IVF clinics monitor every pregnancy. However, extrauterine pregnancies were excluded. Clinical subgroups of patients are summarized in Table 4.

To minimize potential bias related to the lower probability of implantation and successful pregnancy at higher patient age, most patients in this group included in our analysis underwent treatment with donor in vitro fertilization (DIVF) or received genetically tested embryos through preimplantation genetic testing (PGT). Although excluding patients older than 40 years yielded results similar to those observed in the entire study cohort, the overall conclusions of the analysis remained unchanged.

### 4.1. Endometrial Biopsy

Endometrial biopsies were collected between day 19 and 21 of the menstrual cycle, primarily by the Pipelle endometrial sampling (351 patients), and the remainder by curettage. Sampling was performed following sonographic confirmation of the secretory phase. As we confirmed in our previous study, the less invasive Pipelle endometrial sampling is suitable for quantification of uNK cells in patients with RIF and RPL [12]. The routine formalin-fixed and paraffin-embedded technique was used to process the tissue samples, involving the preparation of 4 μm thick histological sections stained with hematoxylin and eosin, as well as with PAS staining, for histopathological assessment of the endometrium.

### 4.2. Immunohistochemical Staining

Tissue sections were examined after immunohistochemical staining. The 4 μm thick tissue sections were boiled in citrate buffer for antigen retrieval. Afterwards, we used antibodies against CD56 (for NK cells), which were visualized using the immunoperoxidase staining technique with a dark-brown product. All chemicals were purchased from Agilent Technologies (Santa Clara, CA, USA) and processed using EnVision FLEX Visualization System in the Autostainer Plus (DAKO, Glostrup, Denmark).

### 4.3. Uterine NK Cell Counting and Interpretation

A single experienced histopathologist manually counted positive cells to minimize the risk of inter-observer variability. Positive cells were counted in three different microscopic fields of 1 mm^2^ at 200× magnification. The results were expressed as the average number of uterine NK cells/mm^2^.

A group of at least 10 uterine NK cells abutting on an endometrial gland was considered a periglandular cluster. The number of periglandular clusters was evaluated as: −, no periglandular clusters; +, one cluster/4 mm^2^ at 100× magnification; ++, two or more clusters/4 mm^2^ at 100× magnification.

### 4.4. Statistical Analysis

We performed a thorough analysis of our collective of 387 patients using ANOVA analysis to find relevant clinical correlations.

## 5. Conclusions

Even though the present paper has not found a direct link between simple uNK cell count change and RIF/RPL risk, we confirmed that uNK cell count is related to their clustering around crucial endometrial structures, which should be the focus of subsequent research endeavors. The uniqueness of this paper lies in that, apart from uNK cell clustering, we analyzed 13 additional variables, including various drugs, food intolerances, and seasonal variations related to uNK cell count. It may seem that the absence of a statistically significant correlation means that our research was futile. We think that it is the complete opposite. By demonstrating that none of these variables correlate with the change in uNK cell count, we helped to set the research direction, which should deemphasize uNK cell quantification and favor the development of clinically translatable methods for assessing the dynamics of the uNK complex.

Both RIF and RM are devastating conditions that profoundly impact couples experiencing infertility. Repeated pregnancy loss leads to emotional distress, psychological suffering, and feelings of helplessness and stigmatization. These issues underscore the unmet medical need for improved diagnostic and therapeutic strategies, while also highlighting the profound social and emotional impact of childlessness. Addressing these conditions is crucial for enhancing reproductive outcomes and providing comprehensive care to affected individuals.

## Figures and Tables

**Table 1:** Statistical correlations of studied variables (factors).

Factor	Correlation	Significance Level (*p*-Value)	Notes
NK Cell Count and presence of clusters	0.161	0.01	Higher NK cell counts correlate with presence of clusters
NK Cell Count and L-thyroxine Treatment	0.014	0.781	No significant correlation found
NK Cell Count and Intralipid Treatment	−0.038	0.459	No significant correlation found
NK Cell Count and Metformin Treatment	0.001	0.980	No significant correlation found
NK Cell Count and Corticosteroid Treatment	−0.011	0.835	No significant correlation found
NK Cell Count and HRT Treatment	−0.015	0.761	No significant correlation found
NK Cell Count and Aspirin Treatment	−0.049	0.340	No significant correlation found
NK Cell Count and LMWH Treatment	−0.010	0.851	No significant correlation found
NK Cell Count and Bromocriptine Treatment	0.012	0.819	No significant correlation found
Impact of Clusters on Pregnancy/Miscarriages	0.031	0.549	No significant correlation found
NK Cell Count by Location (Martin, Bratislava)	−0.053	0.296	No significant association found
NK Cell Count by Season	−0.044	0.393	No significant association found
Impact of food intolerances on NK Cell Count	0.007	0.890	No significant association found

**Table 2:** uNK Cell Counts by Age Group (n = 387).

Age Group (Years)	Number of Patients	Average uNK Cells/mm^2^
20–29	31	94.97
30–39	257	115.55
≥40	99	112.29

**Table 3:** uNK Cell Clusters by Age Group (n = 118).

Age Group (Years)	Patients with Positive Clusters
20–29	9
30–39	70
≥40	39

**Table 4:** Clinical Subgroups.

Clinical Subgroup	Number of Patients
Patients with RIF	218
of which younger than 37	107
Patients with RPL	28
of which younger than 37	16
Patients with idiopathic sterility	158
of which younger than 37	82

## Data Availability

The data presented in this study are available on request from the corresponding author. The data are not publicly available due to concerns that the information could compromise the privacy of research participants.

## Abbreviations

The following abbreviations are used in this manuscript:

ART	Assisted reproductive technologies/treatment
uNK	Uterine natural killer cells
RIF	Recurrent implantation failure
RPL	Recurrent pregnancy loss
ESHRE	European Society of Human Reproduction and Embryology
ASRM	American Society for Reproductive Medicine
RCOG	Royal College of Obstetricians and Gynaecologists
IVF	In vitro fertilization
PGT	Preimplantation genetic testing
HST	Hormone substitution therapy
LMWH	Low molecular weight heparin
EVT	Extravillous trophoblast
PlGF	Placental growth factor
EGF	Epidermal growth factor
IL-6	Interleukin-6
SDF-1	Stromal cell-derived factor-1
CXCL12	CXC motif chemokine ligand 12
CXCR4	CXC chemokine receptor type 4
CXCL8	CXC motif chemokine ligand 8
CCL14	CC motif chemokine ligand 14
CXCL6	CXC motif chemokine ligand 6
ECM	Extracellular matrix
CAM	Cell adhesion molecules
KLRG1	killer cell lectin-like receptor G1
